# Bridging the extracellular vesicle knowledge gap: insights from non-mammalian vertebrates, invertebrates, and early-diverging metazoans

**DOI:** 10.3389/fcell.2023.1264852

**Published:** 2023-08-28

**Authors:** Michaela Liegertová, Olga Janoušková

**Affiliations:** ^1^ Department of Biology, Faculty of Science, Jan Evangelista Purkyně University, Ústí nad Labem, Czechia; ^2^ CENAB, Faculty of Science, Jan Evangelista Purkyně University, Ústí nad Labem, Czechia

**Keywords:** extracellular vesicles, exosomes, cargo, non-mammalian animal models, signalling, regeneration, development

## Abstract

Extracellular vesicles (EVs) are lipid-enclosed structures that facilitate intercellular communication by transferring cargo between cells. Although predominantly studied in mammals, extracellular vesicles are ubiquitous across metazoans, and thus research in non-mammalian models is critical for fully elucidating extracellular vesicles biology. Recent advances demonstrate that extracellular vesicles mediate diverse physiological processes in non-mammalian vertebrates, including fish, amphibians, and reptiles. Piscine extracellular vesicles promote fin regeneration in zebrafish and carry heat shock proteins regulated by stress. Frog extracellular vesicles containing microRNAs modulate angiogenesis, while turtle extracellular vesicles coordinate reproductive functions. Venom from snakes contains extracellular vesicles that mirror the whole venom composition and interact with mammalian cells. Invertebrates also possess extracellular vesicles involved in immunity, development, and pathogenesis. Molluscan extracellular vesicles participate in shell formation and host interactions. Arthropod models, including Drosophila, genetically dissect conserved pathways controlling extracellular vesicles biogenesis and signalling. Nematode extracellular vesicles regulate larval development, animal communication, and ageing via conserved extracellular vesicles proteins. Ancient metazoan lineages utilise extracellular vesicles as well, with cnidarian extracellular vesicles regulating immunity and regeneration. Ultimately, expanding extracellular vesicles research beyond typical biomedical models to encompass phylogenetic diversity provides an unparalleled perspective on the conserved versus specialised aspects of metazoan extracellular vesicles roles over ∼500 million years. With a primary focus on the literature from the past 5 years, this review aims to reveal fundamental insights into EV-mediated intercellular communication mechanisms shaping animal physiology.

## 1 Introduction

Extracellular vesicles (EVs) mediate intercellular communication across the animal kingdom by transferring biomolecular cargo that can alter recipient cell states. First identified in mammals over 40 years ago in rats (Harding et al., 1983), EVs have been extensively characterised in rodents and humans in *vivo* and *in vitro* contexts, providing significant insights into their origin, categorization, distribution, and impacts on immunity or tissue regeneration (van Niel et al., 2018). However, EV research in non-mammalian animals remains limited despite their vast phylogenetic diversity spanning over 500 million years of evolution. This review aims to bridge this knowledge gap by focusing on non-mammalian vertebrates, invertebrates, and early-diverging metazoans, which are often overlooked in EV studies. Examining EVs across this diversity presents an exceptional opportunity to elucidate universally conserved as well as lineage-specific aspects of EV biology adapted to diverse environments.

In general, EVs are membrane bound vesicles encompassing exosomes (Janouskova et al., 2022), microvesicles and other subtypes. They contain proteins, nucleic acids, lipids and other biomolecular cargo that can modulate signalling and function in recipient cells. EVs are produced by all cell types and found in all bodily fluids. They play key roles in health and disease by mediating local and systemic cell-cell communication (van Niel et al., 2018).

Looking beyond typical biomedically relevant models is critical for a comprehensive understanding of the role of metazoan EVs. This review aims to elucidate shared and distinct EV characteristics that were adapted to varying ecologies throughout animal evolution. Integrating discoveries in understudied organisms would provide valuable insights into EV formation, biodistribution, categorization and signalling in multicellular animals. Advancing the rapidly growing field requires prioritising comparative EV biology in diverse, understudied species.

## 2 EVs in non-mammalian vertebrates

While extracellular vesicles (EVs) have been extensively characterised in mammals, studies elucidating EVs in avian species remain scarce. Recent proteomic analysis (Luo et al., 2022) of EVs from rooster seminal plasma identified over 3,000 proteins, including many involved in sperm maturation and antimicrobial protection. This suggests avian EVs may regulate fertility, akin to prostasomes in mammals (Muñoz et al., 2022). Separately, EVs isolated from chicken egg yolks were shown to contain diverse miRNA species (Fratantonio et al., 2023). Feeding studies revealed these egg EVs accumulated in the mouse intestines, brain, and lungs post-ingestion. Egg EV depletion also impaired mouse cognition, while egg consumption increased plasma miRNAs and PBMCs gene expression in humans. Together, these findings provide initial glimpses into the cargo and potential physiological roles of EVs from avian reproductive secretions. Both egg and seminal EVs appear to mediate critical processes in the producer while transferring bioactive molecules to consumers. Further interrogation of the specific bioactive cargoes and functions of these avian EVs is greatly needed. Additional multi-omics profiling and functional validation studies across avian taxa would provide tremendous insights into the conserved *versus* specialised roles of non-mammalian EVs. Elucidating the contributions of these EVs to avian biology and cross-species interaction could uncover innovative approaches to enhance poultry production, egg nutritional value, and human health.

Criscitiello et al. have uncovered extracellular vesicles (EVs) in American alligator plasma, providing the first insights into EV biology in crocodilians (Criscitiello et al., 2020). The characterization of alligator EVs revealed a heterogeneous population with proteomic cargo involved in metabolism, immunity, and epigenetic control. EVs may facilitate moonlighting functions of proteins involved in alligator physiology, like cancer resistance. This establishes a model for elucidating EV mechanisms in ancient reptiles. Expanded EV analysis in diverse reptiles will uncover conserved and unique aspects of their biology.

Work in the Chinese softshell turtle showed biliary EVs originate from hepatocytes, supporting conserved EV roles in cell communication along the biliary system (Zhu et al., 2019). In turtle reproductive tracts, EVs from Sertoli and telocyte sources appear to coordinate spermatogenesis and sperm maturation (Chen and Holt, 2021). Further research into turtle EV cargos and functions will provide evolutionary insights into this signalling mechanism in amniotes.

Proteomic characterization of viper (Carregari et al., 2018) and cobra (Manuwar et al., 2020) venoms revealed the presence of EV-related proteins and cargo, suggesting EVs are an inherent component. Looking beyond venomics, Ogawa et al. (Ogawa et al., 2008) provided the first direct isolation and visualisation of EVs from *Gloydius blomhoffii* snake venom, determining they harbour bioactive cargo that degrades peptides. Expanding on this, Willard et al. (Willard et al., 2021) demonstrated quantitative proteomics of venom EVs can identify biomarkers of envenomation, while another study (Gonçalves-Machado et al., 2022) showed venom EVs containing toxin-processing enzymes are internalised by mammalian cells. Together, these pioneering studies indicate venom EVs may facilitate toxin spread and host effects. Further research on their biogenesis, proteomic content, and pathophysiological roles across diverse snake families is needed to elucidate venom EV biology.

The large fluid compartments in developing Xenopus embryos enabled the isolation of EVs for characterization, revealing dynamic changes in EV composition during embryogenesis (Danilchik and Tumarkin, 2017). Frog thrombocytes were also shown to secrete CD41^+^ EVs containing regulatory miRNAs that can modulate gene expression related to angiogenesis in target cells (Sugimoto and Toume, 2021). Additionally, EVs were isolated from the skin secretions of the amphibian *Bombina maxima* and found to be enriched in proteins involved in the stress response (Wei et al., 2022; Wang et al., 2023). Together, these studies demonstrate that amphibians are a rich source of EVs that likely play important physiological roles. Further research characterising the biogenesis, cargo, and functions of amphibian EVs, both *in vitro* and *in vivo*, promises to provide evolutionary insights into EV biology and intercellular communication in vertebrates. Studies in amphibian models can elucidate the conserved *versus* specialised roles of EVs in development, haemostasis, and innate immunity across species.

Recent studies in zebrafish models have begun to elucidate the roles of extracellular vesicles (EVs) in development, tissue homeostasis, and regeneration *in vivo* (reviewed in Verweij et al., 2019). Zebrafish embryos and larvae provide a powerful system to visualise and track endogenous EVs, revealing their biodistribution and clearance dynamics. Research uncovered tissue-specific EV impacts, like proposed regulation within intestinal epithelia (Bai et al., 2019) and promotion of osteoclast differentiation during bone fracture healing (Kobayashi-Sun et al., 2020). EVs have been tracked in the developing brain (Xu et al., 2017) and heart (Scott, Sueiro Ballesteros et al., 2021), establishing zebrafish as a versatile model to investigate endogenous EVs across development, homeostasis, and disease states.

Exosomes from olive flounder plasma exhibited wound healing and tissue regeneration activities *in vitro* and *in vivo*, accelerating fin regeneration in zebrafish larvae (Jayathilaka et al., 2022). Gene expression analysis indicated immunomodulatory effects, revealing the potential of fish plasma EVs as therapeutic biomaterials. Rainbow trout plasma exosomes were found to be enriched in the heat shock protein Hsp70, which increased after heat stress. However, the stress hormone cortisol reduced Hsp70 in exosomes, highlighting dynamic cargo regulation (Faught et al., 2017). Atlantic halibut serum EVs contained diverse cargo involved in immunity, gene regulation, metabolism, and development (Magnadóttir et al., 2021). Some complement proteins were post-translationally deiminated, including immune factor C3. Selective modification indicates regulated packaging of native and altered proteins via EVs.

Overall, these studies demonstrate the conservation of fundamental EV biology in fish while highlighting its importance in aquatic physiology and tissue repair. Fish provide tractable models to assess EV functions. Understanding piscine EVs has implications for biomedicine, evolutionary biology, and environmental toxicology.

Recent work has identified deiminated proteins and characterised extracellular vesicles (EVs) in the plasma of sea lamprey (*Petromyzon marinus*) (Rast et al., 2021). Proteomic analysis revealed 37 deiminated proteins in lamprey EVs, involved in pathways related to immunity, metabolism, muscle regulation, and cellular stress response. The findings provide the first evidence of post-translational deimination of key immune and regulatory proteins in lamprey plasma and EVs. Research into such an evolutionary ancient organism provides insights into the conserved roles of protein deimination and EVs throughout vertebrate phylogeny.

In nurse sharks (*Ginglymostoma cirratum*), six deiminated proteins were found in plasma, including complement C3, immunoglobulin, and hemopexin (Criscitiello et al., 2019). Plasma EVs, validated by morphology and EV markers, contained three deiminated proteins: novel antigen receptor (NAR), haptoglobin, and hemopexin. Together, this reveals conservation of protein deimination and EV-mediated export as mechanisms for immune regulation in early vertebrates. The study of these phylogenetically distant elasmobranchs provides evolutionary insight into the functional importance of post-translational modifications and EVs.

## 3 Arthropods

Arthropods comprise the most abundant and diverse animal phylum, including insects, arachnids, crustaceans, and other invertebrates. Research across select arthropod models has recently revealed important roles for extracellular vesicles (EVs) in arthropod biology.

In the commercially fished American lobster *Homarus americanus*, EVs isolated from haemolymph were found to range from 10 to 500 nm, with most under 115 nm (Bowden et al., 2020a). Haemolymph proteomics identified deiminated forms of nine proteins involved in metabolism, defence, and the regulation of vesicle release. KEGG pathway analysis revealed that deiminated proteins are involved in viral, bacterial, and fungal defence pathways. The authors suggest lobster EVs and protein deimination may facilitate immune responses and intercellular communication.

EVs isolated from the venom of the Chinese bird spider *Ornithoctonus hainana* ranged from 50 to 150 nm and expressed the EV marker Tsp29Fb (Xun et al., 2021). Proteomics identified 150 proteins related to vesicle transport, virulence factors, neurotoxins, and enzymes such as hyaluronidase. Functional assays showed the EVs exhibit hyaluronidase activity and inhibit human endothelial cell proliferation. The data shed light on the potential contributions of EVs to spider envenomation pathology.

In the tick *Ixodes scapularis*, EVs from salivary glands contained proteins involved in modulating host immunity and physiology (Oliva Chávez et al., 2021). RNAi silencing the EV biogenesis genes vamp33 and synaptobrevin-2 in tick cells reduced EV secretion and impaired tick feeding on mice. The EV-mediated feeding effect depended on modulating dermal gamma-delta T cells. *I. scapularis* EVs also enhanced the transmission of *Anaplasma phagocytophilum* in mice. Tick cell-derived EVs in the 30–200 nm range transmitted the flavivirus Langat virus to human keratinocytes and endothelial cells (Zhou et al., 2018). Cryo-EM imaging verified the purification of exosomes containing viral envelope proteins. The release of EVs from infected tick cells was reduced by the inhibitor GW4869. The authors propose EVs mediate transmission of tick-borne flaviviruses from arthropod to human cells.

The genetic model Drosophila has proven instrumental for elucidating fundamental EV biology, including biogenesis, cargo sorting, and roles in development and physiology (reviewed in Beer and Wehman, 2017). Drosophila EVs comprise exosomes and microvesicles that transport diverse cargos. Comparative analysis found similarities in size, morphology, and RNA contents between fly and human EVs (Lefebvre, Benoit Bouvrette et al., 2016). However, Drosophila EVs were enriched in small nucleolar RNAs, while human EVs contained more Y and vault RNAs. *In vivo* studies revealed EVs carry morphogens like Wingless that pattern tissues during development (Gross et al., 2012). Endocytic trafficking pathways control Wingless sorting into EVs. EVs also mediate physiological changes in the female reproductive tract in response to mating (Sanchez-Lopez et al., 2022). A specific tracheal EV subpopulation associates with fusion events, regulated by Rab GTPases and SNAREs (Camelo, Körte et al., 2022).

Studies in honeybees have revealed therapeutic bioactivities of EVs from bee products (reviewed in Peršurić and Pavelić, 2021). Bee gland secretomal products (honey, royal jelly, and bee pollen) EVs exhibited antimicrobial effects on *Staphylococcus aureus* and were internalised by human mesenchymal stem cells to increase migration (Schuh et al., 2019). Royal jelly EVs contained cargo proteins, and uptake into mammalian cells modulated mesenchymal stem cell differentiation (Álvarez et al., 2023). Anti-inflammatory effects were shown in macrophages, and acceleration of wound closure by royal jelly EVs was confirmed *in vivo*.

Overall, studies in Arthropods establish diverse EV roles in development and physiology. Drosophila enables the systematic dissection of molecular regulators controlling EV functions, providing insights into conserved metazoan communication. Analyses of therapeutic bee EVs warrant further research but show great potential for biomedical applications.

## 4 Molluscs

Molluscs possess a complex system of EVs involved in physiology, immunity, and biomineralization. Proteomic profiling revealed exosomal markers and immune cargo in the haemolymph EVs of four commercially valuable bivalve species (Bowden et al., 2020b). Species-specific variations in yield and size were observed, and deiminated proteins were identified. In freshwater mussels, mantle EVs containing miRNAs were isolated (Chen et al., 2019). Comparative sequencing suggested involvement in shell pigmentation. Mucus from the invasive slug *Arion vulgaris* contained abundant EVs, which could be internalised by plant and human cells and loaded with chemotherapy agents (Liegertová et al., 2022).

These findings demonstrate that molluscs have complex EV communication systems that modulate immunity, biomineralization, and host interactions. Ongoing isolation and sequencing of EVs from diverse mollusc tissues/species is needed to elucidate proteins/RNAs in shell formation. Studying EVs in emerging pests like Arion slugs could reveal novel signalling in plant pathogenesis. Overall, molluscan EVs remain an exciting research frontier at the intersection of marine biology, immunology, materials science, and biotechnology.

## 5 Nematodes

Research utilising the model nematode *C. elegans* has provided significant insights into the mechanisms of EV biogenesis and the functions of EVs in development and physiology. Studies in *C. elegans* demonstrate that both exosomes and microvesicles play critical roles during larval development and adult behaviour. Exosomes containing Hedgehog-related proteins that are essential for proper cuticle formation (Liégeois et al., 2006). The V-ATPase V0 subunit mediates fusion of multivesicular bodies with the plasma membrane for exosome release and secretion of Hedgehog signals. Additionally, ciliated sensory neurons selectively shed EVs in an intraflagellar transport-dependent manner (Wang and Barr, 2016), which could provide valuable insight into human ciliopathies. These neuronal EVs contain polycystins and influence animal-to-animal communication and mating behaviours.

Large-scale proteomic and RNA analysis of nematode EVs identified diverse coding and non-coding RNA and protein cargo types commonly found in human EVs, underscoring the conservation of EV composition (Russell et al., 2020). The EV cargo spectrum suggested that protein and RNA cargoes are actively recruited to EVs. The enrichment for ageing-related factors suggests EVs may modulate longevity and health span. The conservation of EV composition underscores the potential for *C. elegans* to elucidate fundamental mechanisms of EV signalling in ageing.

Recent work found that EVs derived from *C. elegans* dauer larvae, a stress-resistant form, could extend lifespan and health span when fed to normal worms (Ma et al., 2023). The anti-aging effects were associated with reduced oxidative stress and fat accumulation. Together, these findings establish *C. elegans* as a powerful genetic model to elucidate mechanisms of EV biogenesis as well as the functional roles of EVs in development, cell signalling, and ageing. Further exploration of EV biology in this simple organism would provide fundamental insights into conserved metazoan EV pathways.

## 6 Echinoderms

Echinoderm studies have provided insights into multifunctional EVs roles in immunity, regeneration, and signalling. Proteomics of starfish coelomic epithelium revealed numerous exosomal diverse signalling proteins likely participating in immune responses and regeneration abilities (Guatelli et al., 2022). Sea urchin coelomic fluid EVs contained evolutionarily conserved proteins involved in immunity, the cytoskeleton, nuclear functions, metabolism, and stress pathways (D'Alessio et al., 2021). Echinoderm EVs appear to carry both conserved and specialised cargo. For instance, immune proteins underwent post-translational deimination in sea urchin EVs, suggesting regulatory roles in modulating responses. Both studies provided foundational isolation and profiling of echinoderm coelomic fluid EVs to inform future research. Overall, echinoderm EVs reveal intriguing similarities and key specialisations *versus* mammalian EVs. Further analyses across taxa will provide evolutionary perspectives on conserved and specialised EV biology. Studying EVs in regeneration could uncover therapeutic signalling pathways. Unique immune proteins warrant further study for the development of antimicrobial or immunoregulatory biotechnology. Continued echinoderm EV research holds potential for fundamental biological discovery and biomedical innovation.

## 7 Cnidarians

Recent studies demonstrate the presence and importance of EVs as an ancient communication mode in early-diverging metazoans like cnidarians. In the freshwater polyp Hydra, EVs were thoroughly characterised and shown to exhibit exosomal morphology and markers (Moros et al., 2021). Proteomic and RNA sequencing revealed the EVs contained cytoskeletal, extracellular matrix, signalling, and metabolism-related proteins and transcripts, tracing back to multiple cell origins. Excitingly, EVs modulated developmental processes like head and foot regeneration in amputated Hydra. In corals, genes involved in exosome release were upregulated during infection, suggesting exosomes transport immune signals between cells (Takagi et al., 2020). Extracellular matrix genes were also increased, pointing to exosome-ECM interplay in the coral innate immune response.

These studies demonstrate that EVs are an important communication and immune regulation mode that might represent the first language of cell-cell communication to emerge during evolution. Further elucidating the bioactive molecules and physiological functions of cnidarian EVs promises to reveal fundamental metazoan signalling principles. A better understanding of coral innate immunity and EV pathways could support conservation efforts. The emerging picture is that EVs are an ancient language mediating developmental and immune signalling across animal evolution.

## 8 Conclusion

Research across diverse non-mammalian species reveals both highly conserved and uniquely adapted aspects of EV biology. From vertebrates to invertebrates, common themes emerge regarding EVs mediating immune regulation, tissue homeostasis, regeneration, and developmental signalling. Conserved EV biogenic pathways underlie EV release from Hydra to zebrafish. Cargo sorting mechanisms also display conservation, with small RNAs, heat shock proteins, morphogens, and immune factors consistently detected in EVs of organisms separated by hundreds of millions of years. Yet intriguing differences become apparent between mammal and non-mammal EVs. The specific bioactive proteins and RNAs often reflect adaptations to each species’ physiology or environment. Deiminated proteins in ancient vertebrate EVs highlight immune regulation. Enrichment of small nucleolar RNAs in fly EVs contrasts abundance of Y RNAs in human EVs, exemplifying divergence. Unique EVs from nematode neurons demonstrate cell type-specific biogenesis. Moving forward, elucidating EV cargo and impacts across phylogeny remains critical to uncovering new signalling paradigms and biomolecules translatable to biomedicine.
